# Trastuzumab Deruxtecan for Treating HER2-Positive Unresectable or Metastatic Breast Cancer After Two or More Anti-HER2 Therapies: An Evidence Review Group Perspective of a NICE Single Technology Appraisal

**DOI:** 10.1007/s41669-023-00405-2

**Published:** 2023-04-21

**Authors:** Rachel Houten, Nigel Fleeman, James Mahon, Marty Chaplin, Katherine Edwards, Sophie Beale, Angela Boland, Yenal Dundar, Ashley Marsden, Zafar Malik, Carlo Palmieri

**Affiliations:** 1 Liverpool Reviews and Implementation Group, University of Liverpool, Whelan Building, The Quadrangle, Brownlow Hill, Liverpool, L69 3GB UK; 2 Coldingham Analytical Services, Berwickshire, UK; 3 Hare Research, North Yorkshire, UK; 4 North West Medicines Information Centre, Liverpool, UK; 5 The Clatterbridge Cancer Centre, Liverpool, UK; 6 Molecular & Clinical Cancer Medicine, University of Liverpool, Liverpool, UK

## Abstract

The National Institute for Health and Care Excellence (NICE) provides guidance to improve health and social care in England and Wales. NICE invited Daiichi Sankyo to submit evidence for the use of trastuzumab deruxtecan (T-DXd) for treating human epidermal growth factor 2 (HER2)-positive unresectable or metastatic breast cancer (UBC/MBC) after two or more anti-HER2 therapies, in accordance with NICE’s Single Technology Appraisal process. The Liverpool Reviews and Implementation Group, part of the University of Liverpool, was commissioned to act as the Evidence Review Group (ERG). This article summarises the ERG’s review of the evidence submitted by the company and provides an overview of the NICE Appraisal Committee’s (AC’s) final decision made in May 2021. Results from the company’s base-case fully incremental analysis showed that, compared with T-DXd, eribulin and vinorelbine were dominated and the incremental cost-effectiveness ratio (ICER) per quality-adjusted life year (QALY) gained versus capecitabine was £47,230. The ERG scenario analyses generated a range of ICERs, with the highest being a scenario relating to a comparison of T-DXd versus capecitabine (£78,142 per QALY gained). The ERG considered that due to a lack of appropriate clinical effectiveness evidence, the relative effectiveness of T-DXd versus any comparator treatment could not be determined with any degree of certainty. The NICE AC agreed that the modelling of overall survival was highly uncertain and concluded that treatment with T-DXd could not be recommended for routine use within the National Health Service (NHS). T-DXd was, however, recommended for use within the Cancer Drugs Fund, provided Managed Access Agreement conditions were followed.

## Key Points for Decision Makers

**Table:** 

Data were only available for the intervention of interest (trastuzumab deruxtecan [T-DXd]) from a single-arm study, which had immature overall survival (OS) data and a high amount of censoring.
The Evidence Review Group (ERG) identified methodological and clinical issues relating to the matching-adjusted indirect comparisons carried out to compare T-DXd with relevant comparators, and so, uncertain results were generated.
The ERG considered that, as the relative effectiveness of T-DXd versus comparator treatments could not be determined with any degree of certainty, the company’s and the ERG’s cost-effectiveness results were unreliable and should not be used to inform decision-making.
Estimates of OS were highly uncertain, which prevented the National Institute for Health and Care Excellence (NICE) from recommending T-DXd for routine use in the National Health Service (NHS).

## Introduction

The National Institute for Health and Care Excellence (NICE) is an independent organisation that provides national guidance and advice to improve health and social care in England and Wales. The NICE Single Technology Appraisal (STA) process is designed for the appraisal of a single health technology for a single indication, where most of the relevant evidence lies with one company [1]. Typically, the process is used for new pharmaceutical products close to launch. The evidence for an STA is principally derived from a submission by the company that manufactures the technology and is based on a specification developed by NICE. The company’s submission is critiqued by members of the independent Evidence Review Group (ERG), who produce a report.

The NICE Appraisal Committee (AC) considers the submissions from the company, the ERG report and testimony from experts, patients and other stakeholders. Final guidance issued by NICE for cancer drugs can include a recommendation for routine commissioning, managed access via the Cancer Drugs Fund (a source of funding for cancer drugs [2]) or a recommendation to not make the treatment available.

This article presents a summary of the ERG report for the NICE STA of trastuzumab deruxtecan (T-DXd) for treating human epidermal growth factor 2-positive (HER2+) unresectable or metastatic breast cancer (UBC/MBC) after two or more anti-HER2 therapies. Full details of all relevant appraisal documents, including the appraisal scope, ERG report, company and consultee submissions, NICE guidance and comments on each of these, are available on the NICE website [3].

## The Decision Problem

In National Health Service (NHS) clinical practice, treatment options for patients with advanced breast cancer largely depend on a patient’s HER2 status, as well as their hormone receptor status [4]. At the time of Daiichi Sankyo’s company submission (August 2020), the only anti-HER2 therapies recommended by NICE were trastuzumab in combination with paclitaxel for patients with no prior chemotherapy for MBC [5], pertuzumab in combination with trastuzumab and docetaxel for patients with no prior chemotherapy or anti-HER2 therapy for MBC [6] and trastuzumab emtansine (T-DM1) for patients with prior therapy with trastuzumab and/or a taxane [7]. Hence, T-DM1 was most commonly given in the second-line setting. Third-line or later treatment options included eribulin [8], capecitabine [4] or vinorelbine [4], all of which are treatment options for patients regardless of their HER2 status [4, 9].

Although not recommended by NICE, some oncologists also prescribed trastuzumab in combination with chemotherapy after initial treatment with trastuzumab and T-DM1 (i.e. in the third-line setting). Responses provided by 52 centres in England showed that between November 2019 and January 2020 trastuzumab was being prescribed as a third-line treatment in 50% of these centres [10]. This continuation of trastuzumab in these centres was consistent with the European School of Oncology—European Society of Medical Oncology (ESO-ESMO) consensus guidelines [11] that recommend continuing trastuzumab in HER2+ MBC beyond progression on T-DM1. NICE recommended tucatinib in combination with trastuzumab and capecitabine as an option for treating HER2+ locally advanced breast cancer (LABC) or MBC in adults after two or more prior anti-HER2 therapies in April 2022 [12]. As these regimens were not standard care in the NHS at the time of this appraisal, these were not relevant comparators within the scope for this appraisal.

NICE developed a scope for the assessment of T-DXd, in which it was specified that the clinical effectiveness and cost-effectiveness of this treatment should be established within its licensed indication relative to eribulin, capecitabine and vinorelbine, i.e. standard of care in the NHS. The licensed indication for T-DXd for this appraisal was the treatment of patients with HER2+ UBC/MBC after two or more prior anti-HER2 therapies [3]. Five measures of clinical effectiveness were specified in the final scope: overall survival (OS), progression-free survival (PFS), response rate, adverse events (AEs) and health-related quality of life (HRQoL).

T-DXd received a licence extension for the treatment of patients with HER2+ UBC/MBC after one or more prior anti-HER2 therapies in the European Union and UK in July 2022 [13] and August 2022 [14], respectively. This indication for T-DXd was not within the scope for this NICE appraisal.

## Independent Evidence Review Group (ERG) Report

The evidence provided by the company comprised an initial submission, an economic model (which is commercial in confidence) and the company’s response to the ERG’s clarification requests [3]. The ERG report comprised a summary and critical review of the clinical effectiveness and cost-effectiveness evidence provided by the company. The role of the ERG was to:

- Assess whether the company’s submitted evidence conformed to the methodological guidelines issued by NICE
- Assess whether the company’s interpretation and analysis of the evidence were appropriate
- Indicate the presence of other sources of evidence or alternative interpretations of the evidence that could help inform the development of NICE guidance

In addition to providing this detailed critique, the ERG modified a number of key assumptions and parameters within the company’s economic model to examine the impact of such changes on company base-case cost-effectiveness results.

### Clinical Evidence

#### Direct Evidence

The company identified two studies of T-DXd, the phase I DS8201-A-J101 study (*n* = 115) [15] and the phase II, two-part, open-label, multicentre, single-group dose-finding DESTINY-Breast01 [16] (*n* = 184). Both studies evaluated the efficacy and safety of T-DXd in patients with HER2+ UBC/MBC who had received at least two prior anti-HER2 therapies, including trastuzumab and T-DM1. Results from DESTINY-Breast01 [16] were the main source of clinical effectiveness evidence for T-DXd.

In part 1 of DESTINY-Breast01 [16], 65 patients were initially randomised to receive one of three doses of T-DXd administered by intravenous infusion once every 3 weeks: 5.4 mg/kg (*n* = 22), 6.4 mg/kg (*n* = 22) or 7.4 mg/kg (*n* = 21). Following analysis of the pharmacokinetics data, two of these doses were identified for further evaluation in part 1 (5.4 mg/kg and 6.4 mg/kg) and 54 newly enrolled patients were randomly assigned to receive one of the following two doses once every 3 weeks: 5.4 mg/kg (*n* = 28) or 6.4 mg/kg (*n* = 26). The recommended dose of T-DXd (5.4 mg/kg) was then established from a benefit–risk analysis of the data from this study [16] and the phase I DS8201-A-J101 study [15]. A further 134 patients were enrolled into DESTINY-Breast01part 2 [16]. These were patients whose disease had progressed during or after prior therapy with T-DM1 (part 2a: *n* = 130) and patients who had discontinued T-DM1 for reasons other than disease progression (part 2b: *n* = 4). The evidence presented by the company thus comprised 184 patients who had received 5.4 mg/kg of T-DXd (part 1: *n* = 50; part 2: *n* = 134).

The primary objective of DESTINY-Breast01 [16] was to establish the objective response rate (ORR) of patients receiving T-DXd. Secondary outcomes included PFS, OS and safety. The company presented evidence from the August 2019 data-cut (median length of follow-up of 11.1 months, range 0.7–19.9 months). Key DESTINY-Breast01 [16] efficacy and safety results are summarised in Table 1.

**Table 1:** Key results from DESTINY-Breast01, August 2019 data cut-off

Outcome	T-DXd (*n* = 184)	
Primary outcome—ORR		
ICR, *n* (% [95% CI])	112 (60.9% [53.4–68.0])	
Investigator assessment, *n* (% [95% CI])	123 (66.8% [59.5–73.6])	
PFS by ICR		
Events, *n* (%)	58 (31.5%)	
Median PFS, months (95% CI)	16.4 (12.7–NE)	
OS		
Events, *n* (%)	25 (13.6%)	
Estimated OS rate at 6 months, % (95% CI)	93.9% (89.3–96.6)	
Estimated OS rate at 12 months, % (95% CI)	86.2% (79.8–90.7)	
Median OS, months (95% CI)	Not reached	
Treatment emergent AEs, *n* (%)		
Any grade AE	183 (99.5%)	Drug related: 183 (99.5%)
Any grade ≥ 3 AEs	105 (57.1%)	Drug related: 89 (48.4%)
Any serious AEs	42 (22.8%)	Drug related: 23 (12.5%)
AEs leading to T-DXd discontinuation	28 (15.2%)	Drug related: 27 (14.7%)
AEs leading to dose reduction	43 (23.4%)	Drug related: 40 (21.7%)
AEs leading to dose interruption	65 (35.3%)	Drug related: 53 (28.8%)
AEs leading to death	9 (4.9%)	Drug related: 2 (1.1%)

Drug-related AEs are AEs considered by the study investigators to be related to treatment with T-DXd

Source: Company submission [3] and Modi et al. 2020 [16]

*AE* adverse event, *CI* confidence interval, *ICR* independent central review, *NE* not evaluable, *ORR* objective response rate, *OS* overall survival, *PFS* progression-free survival, *T-DXd* trastuzumab deruxtecan

At the time of the DESTINY-Breast01 [16] August 2019 data cut-off, 112 of 184 patients (60.9%) had an ORR by independent central review. In a post hoc subgroup analysis for ORR by number of prior lines of systematic therapy, confirmed ORR was > 50% for each patient subgroup. Median PFS was 16.4 months and median OS had not been reached; estimated OS was 93.9% and 86.2% at 6 months and 12 months, respectively.

All but one of the patients (183/184, 99.5%) in DESTINY-Breast01 [16] experienced a treatment-emergent adverse event (TEAE), which was considered by study investigators to be related to treatment with T-DXd. The most common TEAEs experienced by patients were gastrointestinal or haematological in nature. Approximately half (89/184, 48.4%) of all patients experienced a drug-related grade ≥ 3 TEAE, and 27 of 184 patients (14.7%) discontinued treatment with T-DXd following a drug-related AE. Decreased neutrophil count (38/184, 20.7%) was the only grade ≥ 3 TEAE that was reported in ≥ 10% of patients. The most common drug-related AE that led to discontinuation was pneumonitis/interstitial lung disease (ILD) (9/184, 4.9%). Nine of 184 patients (4.9%) experienced TEAEs that resulted in death; four deaths (2.2%) were attributable to ILD and two (1.1%) were considered to be drug related.

#### Indirect Evidence

In the absence of direct evidence comparing T-DXd to the comparators identified in the NICE scope, the company explored the feasibility of conducting indirect comparisons. As DESTINY-Breast01 [16] was a single-arm study, no connected network to enable a network meta-analysis or a Bucher indirect comparison could be established. Therefore, the company generated indirect evidence using unanchored matching-adjusted indirect comparisons (MAICs) for OS, PFS and response outcomes (ORR, disease control rate [DCR] and clinical benefit rate [CBR]).

The company performed seven MAICs by matching DESTINY-Breast01 [16] data to comparator study data. Four MAICs were presented for T-DXd versus eribulin, utilising data from four studies of eribulin [17–20], two MAICs were presented for T-DXd versus capecitabine, utilising data from two studies of capecitabine [21, 22], and one MAIC was presented for T-DXd versus vinorelbine, utilising data from one study of vinorelbine [23]. The results derived by the company cannot be presented as they are academic in confidence. However, the company concluded that all results demonstrated T-DXd to be associated with an improvement in OS, PFS and response outcomes (ORR, DCR and CBR).

The company considered that the two NICE end-of-life criteria [1] were relevant to this appraisal since: (1) T-DXd is indicated for patients with a short life expectancy (life expectancy for patients with HER2+ MBC is normally less than 24 months) and high unmet need, and (2) T-DXd offers a life extension of at least 3 months versus the life expectancy delivered by the NHS comparator treatment options for these patients.

### Critique of the Clinical Evidence and Interpretation

The ERG considered that the DESTINY-Breast01 [16] population may not appropriately reflect either the population described in the final scope issued by NICE or patients treated in the NHS. Clinical advice to the ERG was that T-DXd would be best placed as a third-line treatment option, following HER2 therapy with trastuzumab (as monotherapy or in combination with pertuzumab and docetaxel) and T-DM1. However, the patients in DESTINY-Breast01 [16] had already received a median of six lines of prior therapy for LABC/MBC, and over half of the patients (54.3%) had received an anti-HER2 therapy that is not recommended by NICE.

Subgroup analysis for patients receiving third-line treatment, for response outcomes, was carried out by the company using pre-specified definitions of lines of prior therapy according to the DESTINY-Breast01 statistical analysis plan (SAP). In addition, results from a post hoc subgroup analysis according to an alternative definition of lines of prior therapy [24] requested by the US Food and Drug Administration (FDA) [25] was also presented. Using both definitions of prior lines of therapy, results were generated using data from small numbers of patients (patients receiving third-line treatment: *n* = 17 [SAP] and *n* = 30 [FDA], respectively). Due to small numbers, the ERG was unable to draw meaningful conclusions from these results.

The ERG highlighted that DESTINY-Breast01 [16] was a single-arm phase 2 study, i.e. there was no relevant comparator, and the reported data were immature (median OS had not been reached and there was a high level of censoring, 159 (86.4%) events). DESTINY-Breast01 [16] PFS data were also heavily censored (126 (68.5%) events); median PFS was reached at a time when only a small number of patients were being followed up. In addition, the ERG highlighted that there were four deaths from ILD, which may be indicative of ILD as an AE of concern.

The ERG also noted issues with the MAICs. The company appropriately considered a number of covariates (potential prognostic factors or effect modifiers) in their MAICs. However, it was not possible to also adjust for HER2+ status and prior anti-HER2 therapy, which the ERG considered were two of the most important variables to adjust for in the MAICs.

Of the comparator studies, only KCSG BR11-16 [23] included people with HER2+ disease who had received prior anti-HER2 therapy. Patients received vinorelbine (with or without lapatinib) in this randomised controlled trial (RCT). In the ERG’s view, this RCT was the only comparator study with a population relevant to this appraisal. However, there were methodological and clinical issues that introduced uncertainty into the MAIC results:

- *Covariates adjusted for*: The ERG highlighted that the six covariates adjusted for in the MAIC using DESTINY-Breast01 [16] and KCSG BR11-16 [23] data were age, Eastern Cooperative Oncology Group Performance Status (ECOG PS; 0/≥ 1), hormone receptor status (positive/negative), number of lines of prior therapy (< 3/≥ 3), prior therapy with pertuzumab (yes/no) and the presence of visceral disease (yes/no). However, other potentially important covariates not adjusted for in this MAIC were prior hormone therapy (yes/no) and brain metastases (yes/no).
- *Prior therapy:* The ERG noted that while patients had received two prior lines of anti-HER2 therapy in KCSG BR11-16 [23], the study authors note that very few patients, if any, had received T-DM1 as one of their prior therapies, as it was not routine practice in South Korea at the time of the trial.
- *Effective sample size (ESS):* The ERG highlighted that the ESS for the weighted DESTINY-Breast01 [11] data for T-DXd was very small (academic in confidence data). Results for OS and PFS were consequently based on very small numbers of events among patients receiving T-DXd. A small ESS is an indication that the weights are highly variable due to a lack of population overlap, and the OS and PFS estimates may therefore be unstable.
- *Statistical interpretation of study results:* The ERG highlighted that the proportional hazards (PH) assumption was violated for both OS and PFS. Therefore, only median survival times rather than hazard ratios (HRs) could be used to compare survival outcomes. As the median OS had not been reached in DESTINY-Breast01 [16], it was not possible to meaningfully compare OS results for T-DXd versus vinorelbine.
- *Validity of results:* The company considered KCSG BR11-16 [23] OS results lacked face validity and were clinically implausible. These conclusions were based on clinical advice received by the company. The company suggested that KCSG BR11-16 [23] OS results were inconsistent with OS results from other studies [17–22], possibly as a result of subsequent treatment(s) received following disease progression on vinorelbine; information relating to subsequent treatment was not reported in the published paper [23].

Regarding the assessment of the two NICE end-of-life criteria [1], the ERG agreed with the company that the life expectancy of patients with HER2+ disease who progress after receipt of T-DM1 as a second-line therapy and are fit enough to receive a third-line treatment is less than 24 months. However, the ERG did not consider the company’s estimates of an OS gain versus any of the three comparators were based on sufficiently robust comparative data to conclude that T-DXd offers an extension to life for these patients.

### Cost-Effectiveness Evidence

The economic evaluation presented by the company compared the cost-effectiveness of T-DXd versus eribulin, versus capecitabine and versus vinorelbine when used to treat patients with HER2+ UBC/MBC after two or more prior anti-HER2 therapies. Using Microsoft Excel, the company constructed a four health-state (progression-free on treatment, progression-free off treatment, progressed and dead) partitioned survival model with a time horizon of 40 years and a cycle length of 1 week. The data sources used to inform the company's choices of parametric models to estimate PFS, OS and time to treatment discontinuation (TTD), along with ERG comment, are presented in Table 2.

**Table 2:** Clinical effectiveness data in the company model

Treatment	Outcome	Data source	Method of estimation	ERG comment
T-DXd	PFS	DESTINY-Breast01 [16]	Log-normal	Log-normal had the lowest AIC and BIC
Eribulin	PFS	MAIC—data from EMBRACE [20]	HR—with additional adjustments made for studies that included HER2− patients from Lv et al. 2018 [27]	MAICs not suitable for decision-making
Capecitabine	PFS	MAIC—data from Fumoleau et al. 2004 [21]	HR—with additional adjustments made for studies that included HER2− patients from Lv et al. 2018 [27]	
Vinorelbine	PFS	Set equal to capecitabine		
T-DXd	OS	DESTINY-Breast01 [16] with HR based on TH3RESA [26] applied over the study period.	Generalised gamma used for extrapolation beyond the study period	The company’s OS projections for T-DXd are unreliable
Eribulin	OS	EMBRACE [20]	Generalised gamma—with additional adjustments made for studies that included HER2− patients from Lv et al. 2018 [27]	A simple between-study analysis unadjusted for patient characteristics without robust ITC techniques, means the validity of the comparator OS estimates is uncertain
Capecitabine	OS	Fumoleau et al. 2004 [21]	Gompertz—with additional adjustments made for studies that included HER2− patients from Lv et al. 2018 [27]	
Vinorelbine	OS	Set equal to capecitabine		
T-DXd	TTD	DESTINY-Breast01 [16]	Exponential	Appropriate as based on clinical advice to the company
Eribulin	TTD	Set equal to PFS		The reliability of this approach is not known
Capecitabine	TTD	Set equal to PFS		
Vinorelbine	TTD	Set equal to PFS		

*AIC* Akaike information criterion, *BIC* Bayesian information criterion, *ERG* Evidence Review Group, *HER2* human epidermal growth factor 2, *HR* hazard ratio, *ITC* indirect treatment comparison, *MAIC* matching-adjusted indirect comparison, *PFS* progression-free survival, *OS* overall survival, *T-DXd* trastuzumab deruxtecan, *TTD* time to treatment discontinuation

The company determined that the log-normal function was the best fit to DESTINY-Breast01 [16] data and used this function to estimate PFS for patients treated with T-DXd. HRs used to generate PFS estimates for patients treated with comparator drugs were estimated using unanchored MAICs generated from EMBRACE [21] data for the comparison of T-DXd versus eribulin, and Fumoleau et al. 2004 [21] data for the comparison of T-DXd versus capecitabine [21]. The Fumoleau et al. 2004 [21] median estimates of PFS for patients treated with capecitabine were similar to KCSG BR11-16 [19] median PFS estimates for patients receiving vinorelbine; therefore, the company assumed that OS and PFS for patients treated with capecitabine and vinorelbine were equivalent.

The company considered that OS data from DESTINY-Breast01 [16] were too immature to be used to populate the model. The company therefore used data from the T-DM1 arm of TH3RESA [26] (a phase III trial of T-DM1 vs physician’s choice) as the basis for modelling OS for patients treated with T-DXd. Patients enrolled in TH3RESA [26] were adults with centrally confirmed HER2+ advanced breast cancer previously treated with anti-HER2 therapies (advanced setting) and a taxane (any setting) and with progression on two or more anti-HER2 therapies, including trastuzumab and lapatinib (advanced setting). The company first calculated an HR for OS for T-DXd in DESTINY-Breast01 [16] versus T-DM1 in TH3RESA [26]. The best fitting function was the generalised gamma function, and this was used to generate long-term OS estimates for patients treated with T-DXd.

OS estimates for patients treated with eribulin and capecitabine were generated by fitting parametric curves to digitised Kaplan-Meier data from EMBRACE [21] and Fumoleau et al. 2004 [21], respectively. The company did not use data from KCSG BR11-16 [23] for patients receiving vinorelbine since the company considered that the OS data from this trial lacked face validity and were clinically implausible.

All patients in DESTINY-Breast01 [16] had HER2+ disease, while several of the comparator studies included a mix of patients with HER2+ and HER2− disease. The company used the HR reported by Lv et al. 2018 [27] to adjust OS and PFS estimates in studies of the comparator treatments that included HER2− patients.

TTD for patients receiving T-DXd was estimated from DESTINY-Breast01 [16] data using an exponential function, and in the absence of TTD data, treatment until progression was assumed for the comparator treatments.

HRQoL data were not collected in DESTINY-Breast01 [16]. Therefore, the company carried out a systematic review of HRQoL (utilities) studies and used published estimates in the model. Based on the approach taken by the company who provided evidence to inform the NICE technology appraisal of eribulin for treating LABC/MBC after two or more chemotherapy regimens (TA423 [8]), ‘progression-free, on treatment’ utility values were calculated as a function of ORR and AE rates; the ORR for patients treated with T-DXd was taken from DESTINY-Breast01 [16], and the ORRs for comparators were taken from the company unanchored MAICs. Baseline utility, tumour response utility and incremental utility of response values were taken directly from TA423 [8] for all treatments (Table 3). Disutilities associated with AEs were also included.

**Table 3:** Summary of utility values for cost-effectiveness analysis

Health state	Intervention	Utility value
PFS	T-DXd	0.750
	Eribulin	0.713
	Capecitabine	0.725
	Vinorelbine	0.717
	SoC	0.713
	Off-treatment	0.704
Progressed	All	0.588

Source: Company submission [3]

*PFS* progression-free survival, *SoC* standard of care, *T-DXd* trastuzumab deruxtecan

Resource use and costs were estimated based on information from DESTINY-Breast01 [16] (usage of primary treatment including the relative dose intensity, and AE rates), based on the cost estimates in TA423 [8] (health state resource use, end-of-life costs and subsequent therapy costs). In the base case, the company assumed 50% drug wastage and that 60% of patients would receive a subsequent therapy. The impact of wastage and subsequent therapy costs were explored in scenario analysis. All costs were inflated to 2018/2019 prices using the inflation indices provided in the Personal Social Services Research Unit (PSSRU) Unit Costs of Health and Social Care [28].

Confidential Patient Access Scheme (PAS) prices (i.e. discounted prices for the NHS agreed with the Department of Health) were in place for T-DXd and eribulin at the time of this appraisal. As the company was unaware of the PAS price for eribulin, the company base-case results only included the PAS price for T-DXd.

The company carried out probabilistic and deterministic sensitivity analyses. The results showed that base-case results were sensitive to the HR applied to TH3RESA [26] data to model T-DXd OS and the selection of the function used to generate OS estimates.

### Critique of the Cost-Effectiveness Evidence and Interpretation

The ERG considered that the modelled patient pathway and the use of a partition survival model structure were appropriate for decision-making. The ERG was satisfied that the algorithms in the company model were accurate. However, the ERG had some concerns with the clinical evidence and the parametric models used to generate survival and TTD estimates (Table 2). The key issues identified by the ERG are detailed below.

The most important comparative clinical effectiveness outcome, from the perspective of generating cost-effectiveness results, was OS. Approximately 95% of the modelled quality-adjusted life year (QALY) gain associated with treatment with T-DXd was driven by gains in OS. However, the ERG considered that the company modelling of OS for patients treated with T-DXd and the comparator treatments using a simple between-study analysis of data was not robust as this approach did not adjust for differences between patient characteristics in DESTINY-Breast01 [16] and TH3RESA [26]. Notable differences between patients enrolled in DESTINY-Breast01 [16] and TH3RESA [26] were the median number of prior therapies, prior therapy with T-DM1, the proportion of the population identified as Asian and the proportion of patients with ECOG PS 0. The ERG considered that the PH assumption did not hold between DESTINY-Breast01 [16] and TH3RESA [26] OS data, which meant that using a non-time variant mortality HR in the model was inappropriate. The only alternative approach to modelling OS for patients receiving T-DXd would be to use results from the company MAICs. The ERG considered that the results of the MAICs were not suitable for decision-making as the populations enrolled in the comparator studies did not wholly match the population described in the final scope.

The utility values used by the company meant that patients receiving treatment with T-DXd had a higher utility in the PFS health state than patients receiving any comparator treatment. The ERG did not consider there to be any evidence to support this assumption. The ERG highlighted that removal of the utility gain for T-DXd in the PFS state would increase the size of the company’s base-case incremental cost-effectiveness ratios (ICERs) per QALY gained for all comparisons.

For patients who responded to treatment and who were still alive at 5 years, the ERG considered that it was unlikely that the monthly oncologist appointment included in the company background healthcare costs would be appropriate; rather there would be longer periods between appointments. Excluding the monthly oncologist cost would reduce the total background health state costs for this group of patients. If treatment with T-DXd extended life more than the comparator treatments (as claimed by the company), and if long-term health state costs that the ERG considered more appropriate were used, the size of the company’s base-case ICERs per QALY gained would be decreased for all comparisons.

In the company model, a lifetime duration of treatment effect was assumed. However, there is an absence of evidence to support or refute this assumption. The ERG considered that if the treatment effect did not last a patient’s lifetime (i.e. mortality and progression hazards become equal for all treatments at a future point in time), the size of the ICER per QALY gained would be increased for the comparison of T-DXd versus all the comparators.

### Conclusions of the ERG report

The ERG considered that the company was unable to provide evidence that allowed a robust comparison of the clinical effectiveness of T-DXd versus any of the relevant comparators for the following reasons:

- DESTINY-Breast01 [16] was a phase II, single-arm study with immature data;
- Results from the MAICs comparing T-DXd versus eribulin and T-DXd versus capecitabine related to populations that were not relevant to the decision problem;
- As the PH assumption was violated in the MAIC comparing T-DXd versus vinorelbine, and as median OS had not been reached in DESTINY-Breast01 [16], a meaningful comparison of T-DXd versus vinorelbine for this outcome could not be made.

The ERG considered that as the relative clinical effectiveness of T-DXd versus the comparator treatments could not be determined with any degree of certainty, the company cost-effectiveness results were unreliable and should not be used as the basis for decision-making.

## Technical Engagement

A key aim of the technical engagement process is to help the NICE AC to make recommendations at the first NICE AC meeting, i.e. without the need for subsequent meeting(s). Within 30 days of the ERG report being submitted to NICE, the technical engagement papers are sent to consultees and the company for comment.

Eight key issues were identified by the ERG, and three issues were raised by the NICE technical team. Prior to the first NICE AC meeting, the company presented a response to these comments. The company response included new clinical effectiveness evidence from a MAIC comparing T-DXd versus capecitabine, utilising capecitabine data from EGF100151 [29], a trial not previously included in the company’s submission. EGF100151 [29] was a phase III RCT comparing lapatinib plus capecitabine versus capecitabine in women who progressed after regimens that included an anthracycline, a taxane and trastuzumab. The company also presented new base-case and scenario analysis cost-effectiveness results using updated OS data for T-DXd from DESTINY-Breast01 [30]. The ERG was asked to respond to the comments submitted by the company. The identified issues, along with the company and ERG responses, are summarised in Table 4.

**Table 4:** Issues raised at technical engagement

Issue	Company response	ERG response
ERG 1: Immature DESTINY-Breast01 [16] data	Presented more mature data (median follow-up of 20.5 months, to June 2020, with 35% of death events occurring and a median OS of 24.6 months [95% CI 23.1–NE]) from DESTINY-Breast01 [30]	DESTINY-Breast01 [30] data were still immature, but the additional follow-up time meant that median PFS and median duration of response results were more robust than those originally presented
ERG 2: Lack of direct effectiveness evidence for the comparison of T-DXd versus relevant comparators (eribulin, capecitabine and vinorelbine)	When completed, an ongoing phase III trial, DESTINY-Breast02 [34], will provide direct evidence for the effectiveness of T-DXd versus ‘investigator’s choice’ (anti-HER2 therapy [trastuzumab or lapatinib] in combination with capecitabine). However, investigator’s choice options are not relevant comparators in this appraisal	No additional comment
ERG 3: Due to the number and type of prior lines of therapy received by patients enrolled in DESTINY-Breast01 [16], the relevance of DESTINY-Breast01 results to NHS clinical practice is unclear	DESTINY-Breast01 [16] subgroup results suggested that ORR was higher in patients who received 2 lines of prior therapy compared with those who received > 2 lines of therapy. Furthermore, results from EGF100151 [27], a trial of patients who had received ≥ 1 prior anti-HER2 therapy, show TTP at earlier lines of therapy is longer than at later lines of therapy in an HER2+ MBC population treated with capecitabine or lapatinib in combination with capecitabine	These results suggested that patients who had received 1 line of prior therapy may have better efficacy than those who had received ≥ 2 lines of prior therapy. However, as patients who had only received 1 prior line of therapy had longer ORR and TTP than patients who had received ≥ 2 lines of prior therapy, this reinforced the importance of including patients in the MAICs who had received ≥ 2 prior anti-HER therapies
ERG 4: Company eribulin and capecitabine MAIC results are not suitable for decision-making	Updated MAICs with the June 2020 data-cut from DESTINY-Breast01 [30] and presented a new MAIC for capecitabine, which included data from EGF100151 [27], a trial of patients who had received ≥ 1 prior anti-HER2 therapy. All MAIC results are academic in confidence	This MAIC was more relevant to the current appraisal than the capecitabine MAICs previously presented. However, ideally, only results from those patients who had received ≥ 2 lines of prior anti-HER2 therapy should have been included in this analysis. Additional issues with the new analysis were a lack of clarity as to which patients were included in the PFS and OS analyses and insufficient information to validate the MAIC data inputs that generated results for ORR, DCR and CBR
ERG 5: Company vinorelbine OS MAIC results are inconclusive	Updated DESTINY-Breast01 [30] data suggested that the PH assumption no longer appeared to be violated for OS data for T-DXd versus vinorelbine. The PH assumption was still violated for PFS data, so updated PFS results were based on accelerated failure time models, which suggested that T-DXd was associated with statistically significantly longer PFS than vinorelbine	Utilising the more recent data-cut from DESTINY-Breast01 [30] alleviated the concern regarding the PH assumption in relation to OS data. However, the concerns regarding covariates that had not been adjusted for and the small ESS for the weighted DESTINY-Breast01 data remained valid areas of uncertainty for both OS and PFS outcomes
ERG 6: Company OS modelling of T-DXd is not robust	An exploratory scenario analysis in which OS data from the June 2020 data-cut of DESTINY-Breast01 [30] were directly extrapolated. Cost-effectiveness results using the average of the Weibull and exponential curves showed that, compared with T-DXd, eribulin and vinorelbine were dominated and the ICER per QALY gained versus capecitabine was £49,028. DESTINY-Breast01 [30] OS data were still too immature to robustly estimate long-term OS for patients receiving T-DXd with any reasonable degree of certainty	Queried whether direct extrapolations were conducted as the data presented appeared to display parametric OS curves generated by applying an HR to TH3RESA [26] OS data and not direct extrapolations from DESTINY-Breast01 [30]. As noted in response to ERG 1, the company considered that the data from the June 2020 data-cut were still too immature to be used directly to estimate OS for the 40-year model time horizon. Thus, it followed that the available data were also too immature to estimate HRs between T-DXd and comparator treatments
ERG 7: Company OS modelling of comparator treatments is not robust	Modelling approach represented a naïve comparison between T-DXd and the modelled comparators. The only alternative approach would be to use the results of the MAICs	Agreed with the company that this was a naïve comparison. As the MAIC results were uncertain, it was not possible to determine whether the naïve approach generated optimistic or pessimistic OS projections for the comparators
ERG 8: NICE end-of-life criteria [1] may not be met in terms of the life extension of 3 months beyond current estimates of survival for all three comparators	Referred to evidence from previous technology appraisals [6, 7] and published literature for eribulin [12–15], capecitabine [16, 17] and vinorelbine [18]. A naïve comparison of the data with updated DESTINY-Breast01 [28] trial data (median OS of 24.6 months) shows that life extension with T-DXd is greater than 3 months (highest median OS in trials of a relevant comparator was 18.9 months in KCSG BR11-16 [23])	No additional comment
NICE 1: Provide updated analyses with the latest data-cut from DESTINY-Breast01	Updated analyses from DESTINY-Breast01 [30], which had more mature data, were presented based on applying an HR to T-DM1 (and not the exploratory scenario analysis described in response to ERG 6). Results from the company’s base-case fully incremental analysis showed that, compared with T-DXd, eribulin and vinorelbine were dominated and the ICER per QALY gained versus capecitabine was £47,230	Confirmed that the model provided at technical engagement correctly implemented the changes as described by the company. Also confirmed that the results presented by the company could be reproduced by this model
NICE 2: Explain how these data can lift some of the uncertainties raised by the ERG	In updated analyses from DESTINY-Breast01 [30], preliminary median OS was now reached and estimates of median PFS and duration of response were more certain	Not possible to generate more plausible cost-effectiveness results since OS data were still immature. In particular, the uncertainty around long-term OS for patients receiving T-DXd meant the ICERs per QALY gained generated by the company were likely to be optimistic
NICE 3: Would additional data collection in the CDF, alongside the ongoing DESTINY-Breast02 [34] RCT, reduce the uncertainty?	DESTINY-Breast02 [34] would be the primary source of additional data, with data collection in the CDF being only supportive	No additional comment

*CBR* clinical benefit rate, *CDF* Cancer Drugs Fund, *CI* confidence interval, *DCR* disease control rate, *ERG* Evidence Review Group, *ESS* effective sample size, *HER2* human epidermal growth factor 2, *HR* hazard ratio, *ICER* incremental cost-effectiveness ratio, *MAIC* matching-adjusted indirect comparison, *MBC* metastatic breast cancer, *NE* not evaluable, *NHS* National Health Service, *NICE* National Institute for Health and Care Excellence, *ORR* objective response rate, *OS* overall survival, *PFS* progression-free survival, *PH* proportional hazards, *QALY* quality-adjusted life year, *RCT* randomised controlled trial, *T-DM1* trastuzumab emtansine, *T-DXd* trastuzumab deruxtecan, *TTP* time to treatment progression

## National Institute for Health and Care Excellence Guidance

In addition to the evidence presented by the company and included in the ERG critique, the NICE AC considered the views of patients and clinical experts. Following discussions with the patient and clinical experts, the company and the ERG at the NICE AC meeting, the NICE AC made several observations. The key points raised are summarised below.

### Consideration of the Clinical Effectiveness Issues

The NICE AC considered that the clinical evidence from DESTINY-Breast01 [16, 30] was relevant to UK NHS practice. Although the majority of patients in the study had received more than two lines of prior therapy, the NICE AC considered that as efficacy usually decreases with each additional line, results from the study were likely to underestimate the efficacy of T-DXd in a population that had received fewer prior therapies.

The NICE AC considered that conclusions that could be drawn from DESTINY-Breast01 [16, 30] data were limited due to the single-arm design and the immaturity of the OS data. However, the clinical experts advised the NICE AC that despite the limitations of the OS data, the T-DXd ORR and median PFS in DESTINY-Breast01 [16] were higher than the ORRs and median PFS associated with other anti-HER2 therapies studied in this population [26, 31–33]. The NICE AC therefore considered that although the clinical efficacy results were promising, they were limited in their ability to directly inform decision-making.

The NICE AC considered that the MAIC results showed that T-DXd was associated with improved ORR, PFS and OS compared with eribulin, capecitabine and vinorelbine, but there were major limitations to the MAIC methods. Primarily, HER2 status and prior anti-HER2 therapy could not be adjusted for and not all patients had HER2+ disease or at least two prior lines of anti-HER2 therapy in the eribulin trial (EMBRACE) [20]. While EGF100151 [29] included a population with HER2+ disease treated with capecitabine, there was still some uncertainty in the results from the MAIC because while patients had received one prior line of anti-HER2 therapy in EGF100151 [29], not all patients had received two prior lines. The NICE AC also noted that the analysis only included patients who did not cross over to receive lapatinib plus capecitabine after disease progression on capecitabine, which may have introduced selection bias. The NICE AC noted that KCSG BR11-16 [23] included patients treated with vinorelbine that matched the DESTINY-Breast01 [16] population in terms of HER2 status and having received two prior lines of anti-HER2 therapy. However, clinical experts explained that as it was a small RCT conducted in South Korea, its generalisability to UK clinical practice was unclear. The clinical experts suggested more meaningful comparisons could be conducted using control arms from more recent trials for anti-HER2 therapies [26, 31–33], even though they do not represent current NHS practice. The NICE AC concluded that although the MAICs have limitations, it is likely that T-DXd improves clinical outcomes, compared with eribulin, capecitabine and vinorelbine, but that the magnitudes of the effects were uncertain.

The NICE AC noted that treatment with T-DXd is often associated with ILD. The clinical experts advised that there are other NICE-approved treatments that can cause this AE. Thus, clinical teams have experience of appropriate monitoring for signs and symptoms and providing early treatment to avoid any long-term problems. The NICE AC considered that the AE profiles of non-targeted chemotherapies were also potentially problematic. Therefore, the NICE AC concluded that the safety profile of T-DXd was acceptable.

### Consideration of the Cost-Effectiveness Issues

The NICE AC considered that the company’s cost-effectiveness model generated results that could be used to inform decision-making. However, the NICE AC considered the company’s approach to modelling PFS to be uncertain as the PFS estimates for the comparator treatments were based on the HRs produced by the MAICs, which the NICE AC considered produced unreliable results. Additional uncertainty related to TTD for comparator treatments which, in the absence of TTD estimates from the clinical studies, were assumed by the company to equal PFS.

The NICE AC noted that in the company technical engagement base case, OS was modelled using 20.5 months follow-up DESTINY-Breast01 [30] OS data, followed by a generalised gamma distribution fitted to TH3RESA [26] T-DM1 OS data. In the company technical engagement base-case fully incremental analysis, eribulin was dominated and vinorelbine was extendedly dominated by T-DXd. The NICE AC considered that the company technical engagement base-case ICER for the comparison of T-DXd versus capecitabine (£47,230 per QALY gained, generated using the confidential PAS discounted price of T-DXd) was not implausible but was highly uncertain given the uncertainty around reliability of the clinical effectiveness data used to populate the model. The ERG scenarios generated a range of ICERs (up to £78,142 per QALY gained for T-DXd versus capecitabine), which the NICE AC also considered to be uncertain, but not necessarily implausible, given the high degree of uncertainty surrounding the clinical effectiveness evidence.

In addition to considering that the naïve comparisons produced estimates of OS that were too uncertain to inform decision-making, the NICE AC also considered that the MAICs were too uncertain to inform decision-making. The NICE AC concluded that an RCT of T-DXd was required, which would at least enable an indirect treatment comparison via a connected network of the comparator arm. The NICE AC considered any future modelling of OS should be based on more mature T-DXd data than currently available, rather than utilising data from the T-DM1 arm of TH3RESA [26].

The NICE AC considered that the company’s approach to estimating utility was broadly appropriate, but highlighted some areas of uncertainty. These included the fact that estimates for patients receiving T-DXd were derived from the TA423 [8] appraisal (as no applicable HRQoL data were collected as part of DESTINY-Breast01 [16, 30]) and that the utility estimates associated with the comparator treatments were derived from the MAICs, which have limitations.

The NICE AC agreed with the company that the life expectancy of patients with HER2+ UBC/MBC that have been treated with two or more prior anti-HER2 therapies is less than 24 months. The NICE AC considered that, although highly uncertain given the immaturity of even updated DESTINY-Breast01 [30] OS data, T-DXd was likely to provide an extension to life of at least 3 months. The NICE AC considered that the NICE end-of-life criteria [1] were met. The NICE AC therefore concluded that the ICER could be higher than accepted cost-effectiveness thresholds, even with end-of-life criteria [1] considered.

### Final Guidance

The final guidance was published by NICE in May 2021 [3]. The NICE AC did not recommend T-DXd after two or more anti-HER2 therapies as a routine treatment option for adult patients with HER2+ UBC/MBC. However, T-DXd was recommended for use as an option for treating HER2+ UBC/MBC in adults who have received two or more prior anti-HER2 therapies within the Cancer Drugs Fund. It is recommended only if the conditions in the Managed Access Agreement (MAA) are followed; these conditions are specified in the MAA document published on the NICE website [3]. In summary, T-DXd will be available to the NHS at a reduced cost via the Cancer Drugs Fund while additional data are being collected. The MAA includes details of who can be treated with T-DXd via the Cancer Drugs Fund, the specific trial outcome data to be collected and the data sources that are expected to inform the company’s updated evidence submission and updated economic model (scheduled to be submitted to NICE Q4, 2023). This Cancer Drugs Fund review will determine whether T-DXd can be recommended as a routine NHS treatment option.

## Conclusions

For this appraisal, the NICE technical engagement process enabled the NICE AC to make recommendations without the need for a second NICE AC meeting. However, a number of issues were still unresolved following the technical engagement process and NICE AC meeting. The key clinical effectiveness issues arose from the difficulty in comparing treatment with T-DXd versus a comparator treatment used routinely in NHS clinical practice. This was primarily because of the lack of a head-to-head RCT, the presence of important population differences between the studies included in the MAICs, and the key statistical assumption for PH being violated for OS.

Despite making modifications to the company’s economic model and generating estimates for the ICER per QALY gained for a comparison of the cost-effectiveness of T-DXd versus eribulin, capecitabine and vinorelbine, the ERG considered that the ICERs per QALY gained generated by its modifications should only be considered as exploratory. Primarily, this was because all the estimates depended on the reliability of available clinical effectiveness evidence, and the ERG considered the relative effectiveness of T-DXd versus the comparators could not be determined with any degree of certainty.

## Declarations

### Availability of data and material

Additional information can be found on the NICE website for this topic: https://www.nice.org.uk/guidance/ta704
